# Genetic Evidence for Hybrid Trait Speciation in *Heliconius* Butterflies

**DOI:** 10.1371/journal.pgen.1000930

**Published:** 2010-04-29

**Authors:** Camilo Salazar, Simon W. Baxter, Carolina Pardo-Diaz, Grace Wu, Alison Surridge, Mauricio Linares, Eldredge Bermingham, Chris D. Jiggins

**Affiliations:** 1 Department of Zoology, University of Cambridge, Cambridge, United Kingdom; 2 Smithsonian Tropical Research Institute, Balboa, Panamá; 3 Instituto de Genética, Universidad de los Andes, Bogotá, Colombia; University of Arizona, United States of America

## Abstract

Homoploid hybrid speciation is the formation of a new hybrid species without change in chromosome number. So far, there has been a lack of direct molecular evidence for hybridization generating novel traits directly involved in animal speciation. *Heliconius* butterflies exhibit bright aposematic color patterns that also act as cues in assortative mating. *Heliconius heurippa* has been proposed as a hybrid species, and its color pattern can be recreated by introgression of the *H. m. melpomene* red band into the genetic background of the yellow banded *H. cydno cordula*. This hybrid color pattern is also involved in mate choice and leads to reproductive isolation between *H. heurippa* and its close relatives. Here, we provide molecular evidence for adaptive introgression by sequencing genes across the *Heliconius* red band locus and comparing them to unlinked wing patterning genes in *H. melpomene*, *H. cydno*, and *H. heurippa*. 670 SNPs distributed among 29 unlinked coding genes (25,847bp) showed *H. heurippa* was related to *H. c. cordula* or the three species were intermixed. In contrast, among 344 SNPs distributed among 13 genes in the red band region (18,629bp), most showed *H. heurippa* related with *H. c. cordula*, but a block of around 6,5kb located in the 3′ of a putative *kinesin* gene grouped *H. heurippa* with *H. m. melpomene*, supporting the hybrid introgression hypothesis. Genealogical reconstruction showed that this introgression occurred after divergence of the parental species, perhaps around 0.43Mya. Expression of the *kinesin* gene is spatially restricted to the distal region of the forewing, suggesting a mechanism for pattern regulation. This gene therefore constitutes the first molecular evidence for adaptive introgression during hybrid speciation and is the first clear candidate for a *Heliconius* wing patterning locus.

## Author Summary

Hybrid speciation challenges our view of biodiversity as a branching tree and is considered rare or absent in animals. A possible route by which it may occur is establishment of a novel “magic trait,” influencing both ecological adaptation and mating preference, via hybridization. We provide, to our knowledge, the first molecular genetic evidence for this process in the tropical butterfly *Heliconius heurippa*. We sampled molecular markers both linked to the locus controlling red color pattern and across the genome of *Heliconius heurippa* and its putative parents, *H. cydno* and *H. melpomene*. We found evidence of genetic introgression from *H. melpomene* into the hybrid *H. heurippa* only at the genomic region of the forewing red-band locus. This signature of introgression corresponds to the 3′ end of a *kinesin* gene that also shows a pattern of expression restricted to the distal region of the forewing. As the wing color pattern in these butterflies is crucial in maintaining the isolation of this species through mate choice, this study provides molecular support for the hybrid origin of a new adaptive trait that can lead to speciation.

## Introduction

Identifying the genetic mechanisms involved in speciation is an important challenge in the study of evolution [1]–[3]. Empirical studies have shown that species differences can be localized in just a few genomic regions [3]–[5], and that reproductive isolation is more easily achieved when traits causing assortative mating are also subject to natural selection [6], [7]. Such characteristics have been termed ‘magic traits’ [6] and can facilitate speciation as a side-effect of ecological divergence in the presence of ongoing gene flow [8], [9]. Likely examples of such magic traits include body size and color in sticklebacks, flowering time in edaphic plants, host shifts in phytophagous insects, color patterns in *Heliconius* butterflies, beak size in Darwin finches, development time in melon flies and color patterns in *Hypoplectrus* fish [9]–[15].

If ‘magic traits’ were acquired by introgression from related lineages, adaptation and speciation could proceed without the requirement for novel mutations [16], [17]. Recent studies in plants and animals have shown that introgression can provide the raw material for adaptation [18]–[20]. Hence, it is plausible that if introgression produces new adaptive phenotypes that also generate reproductive isolation, for example through mate choice, habitat colonization or asynchronous emergence, then hybrid speciation can occur without geographic isolation [17], [21]. We have called such a scenario ‘hybrid trait speciation’, as a special case of speciation through hybridization without a change in chromosome number or homoploid hybrid speciation (HHS) [2], [22]. Hybrid trait speciation contrasts with what we have termed mosaic genome speciation, documented in *Helianthus* sunflowers, where the hybrid species genome is composed of blocks derived from both parental species [23]. Rapid establishment of incompatibilities between parental and daughter species can result due to the large number of genes causing epistatic hybrid breakdown in hybrids [23]. The two scenarios contrast in their genomic signature, with hybrid trait speciation potentially involving introgression of just a few adaptively important loci into the genetic background of one of the parental species, making it much more difficult to detect using traditional approaches based on ‘neutral markers’ [21]. This is in addition to the fact that detecting hybrid speciation at the molecular level is difficult anyway, due to incomplete linage sorting and historical gene flow, which can leave similar signatures of shared variation [24].

There is evidence that hybridization has played an important role in the adaptive radiation of *Heliconius* butterflies [21]. *Heliconius* have aposematic wing coloration, mimicry between divergent species and frequent hybridization, providing an excellent opportunity to study the genetics of adaptation and speciation [25]–[28]. In particular, studies in the closely related species *H. melpomene* and *H. cydno*, that occur sympatrically throughout Central America and in the west Andes, show that mimicry shifts are coupled with assortative mating and lead to speciation [29]. In addition, differences in host plant use, microhabitat preferences and partial hybrid sterility also contribute to reducing genetic interchange between these species [30]. The species hybridize in both the field and the laboratory, although natural interspecific hybrids are collected at a very low rate (one in a thousand or less) [28], [30]. Nonetheless, introgression of color pattern alleles has been observed in natural hybrid zones and the same phenotypes have been recreated in experimental crosses [31], [32]. Furthermore, studies using neutral markers reveal that introgression between the species has been frequent throughout their evolutionary history [33], [34].

Occasionally, novel color pattern variants produced through hybridization appear to have produced stable hybrid populations. The best studied example is *Heliconius heurippa*, found in the eastern slopes of Colombian Andes, which has a color pattern that can be recreated in the laboratory through crosses between *H. c. cordula* and *H. m. melpomene*, the races of the melpomene group adjacent to its current geographical range [35], [36]. *H. heurippa* is abundant and its color pattern is stable along several hundred kilometers of the Andean slopes, although is not mimetic with any other species [30], [37]. This wing pattern stability across a broad geographic area contrasts with the transient production of hybrid forms in narrow *Heliconius* hybrid zones [25].

Surprisingly, only three generations of crosses are needed to obtain a homozygous *H. heurippa* like color pattern [35]. Two tightly linked loci controlling the red forewing band (B allele, hereafter *HmB*) and the absence of brown forceps marks in the ventral surface (br allele) from linkage group 18 are introgressed into an *H. c. cordula* genetic background that includes the yellow forewing band allele at the *HmN* locus on linkage group 15. The resultant pattern contributes to reproductive isolation through assortative mating, and therefore plays a direct role in speciation [35]. In particular, mating experiments revealed strong pre-mating mating isolation between *H. heurippa* and *H. melpomene* (≈90%) and between *H. heurippa* and *H. cydno* (≈75%). Furthermore, assays with wing models showed that *H. heurippa* males use the combined red and yellow bands to discriminate females [35]. Even first-generation backcross hybrids between *H. m. melpomene* and *H. c. cordula*, resembling *H. heurippa*, showed a strong preference for their own color pattern over that of either parental species, implying that mate preference can be established directly through hybridization [38]. This result implies, first that assortative mating preferences would facilitate the initial establishment of a homozygous hybrid color pattern by increasing the probability that early generation hybrids mate among themselves. Second, once the new hybrid population was established, it would immediately possess the assortative mating preferences that generate partial reproductive isolation from the parental species. Thus, *H. heurippa* is an excellent candidate for speciation through adaptive introgression [38].

Despite extensive support for the hybrid origin of *H. heurippa* from biogeography, crosses, mate choice experiments and mathematical simulations [39], molecular evidence for a hybrid origin is inconclusive. Neutral markers reveal extensive gene flow between all three species, *H. heurippa*, *H. melpomene* and *H. cydno* [40]. To definitively test the hybrid origin hypothesis we need to study the loci controlling the adaptive traits responsible for speciation, in this case wing patterns [21], [40]. Here, we take advantage of the recent cloning of the *HmB* locus controlling the red forewing band of *H. melpomene*, in order to carry out such a test [41], [42].

## Results

Previous work on *H. heurippa* has used a panel of largely intronic markers and shown evidence for ongoing gene flow between *H. heurippa* and its close relatives [40]. Here, we directly addressed the adaptive introgression hypothesis by sampling 18 contigs based on 24 amplicons representing 18,629bp across the *HmB* locus for ten individuals of each species (60 alleles; Table S1) [41], [42]. In addition, we improved our broader genome sampling by developing a larger panel of unlinked molecular markers based on single copy genes with large exons. We analyzed around 15,000 contigs assembled from *H. melpomene* GSS (BAC-end) sequences, 484 of which showed strong homology with single copy genes in *B. mori*. From these unigenes, 27 had exons longer than 700 bp (Table S2). Additionally, we used two previously published markers, CAD and GAPDH [43]. Thus, we used a set of 29 markers that were putatively distributed in at least 17 of the 21 chromosomes in *H. m. melpomene* (Table S2). Sequences of these markers were obtained for the same ten individuals per species used in the *HmB* locus analysis (60 sequences per gene).

**Figure 1:**
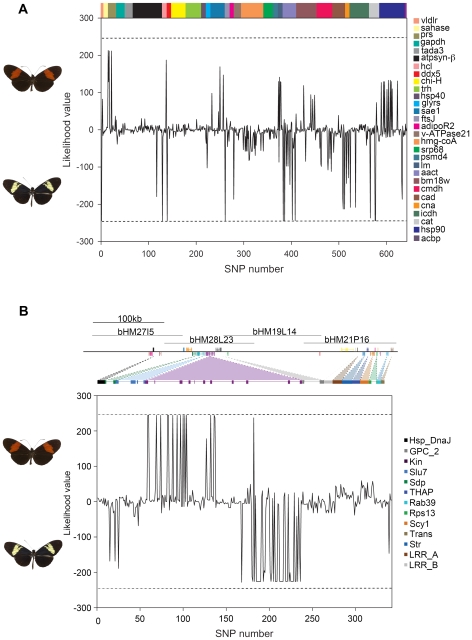
Relative likelihood of association between *H. heurippa* and *H. m. melpomene* versus *H. c. cordula* for (A) genes unlinked to color pattern and (B) across the *HmB* red color pattern locus. Likelihood values are plotted for each variable position, where positive likelihood values indicate a SNP position at which *H. heurippa* and *H. m. melpomene* are more similar, and negative values a position at which *H. heurippa* shows a stronger association with *H. c. cordula*. Fixed sites are indicated by dotted lines showing a likelihood value of 244 for a complete association of *H. heurippa* with *H. m. melpomene* and −244 for that with *H. c. cordula*. Colors represent different coding regions. The majority of unlinked SNPs (634) show shared polymorphism among the three species (240> ΔLnL >−240). At unlinked loci, *H. heurippa* and *H. c. cordula* shared fixed polymorphism at only 8 SNPs whereas *H. heurippa* and *H. m. melpomene* did not share any fixed polymorphism. For the *HmB* locus, sequenced BAC clones are indicated above the gene annotation [41].

Among the 29 loci sampled from across the genome, most SNP polymorphisms were shared among the three species and therefore did not associate *H. heurippa* with one or other of the two parental species. Only 8 SNPs (over six genes) were fixed polymorphisms shared by *H. heurippa* and *H. c. cordula* relative to *H. m. melpomene* (Figure 1A), consistent with previous data from mitochondrial genes that related *H. heurippa* with *H. cydno* [40], while no SNPs were fixed in *H. heurippa* and *H. m. melpomene* relative to *H. c. cordula* (Figure 1A).

Our prediction derived from the adaptive introgression hypothesis was that within the *HmB* locus there should be a region introgressed from *H. melpomene* into the *H. heurippa* genome. This prediction was upheld. From nearly 19Kb of sequence analyzed across the *HmB* locus, there was a 6,493 bp region corresponding to the 3′ end of a putative *kinesin* gene (hereafter, 3′ *kinesin*) showing a strong association between *H. heurippa* and *H. m. melpomene* (Figure 1B). Across the remaining *HmB* region there was either shared variation among the three species, unique polymorphisms in one of the three species, or nearly fixed changes unique to *H. heurippa*. In the case of two genetic markers, *kin_2* and *sdp*, *H. heurippa* was most strongly associated with *H. c. cordula* (Figure 1B).

**Figure 2:**
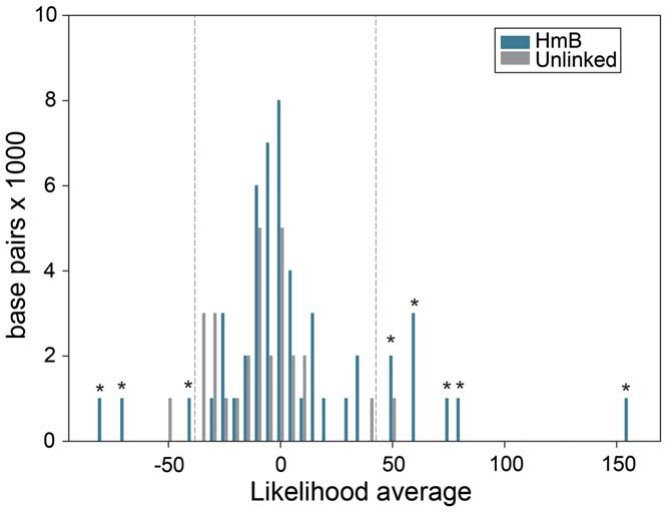
Distribution of average likelihood values at SNPs in unlinked and *HmB* linked loci. The average of likelihood values for each unlinked marker and for 1,000 bp windows in the *HmB* region was calculated. This histogram shows the distribution of these values. Dotted lines represent the 95% two-tailed interval for unlinked genes. The asterisk over the bars indicates those 1,000 bp windows showing average values that lie outside the unlinked genes distribution (p<0,005). Positive values outside that distribution correspond to *3′ kinesin* whereas negative values are those in *kin_2*.

**Figure 3:**
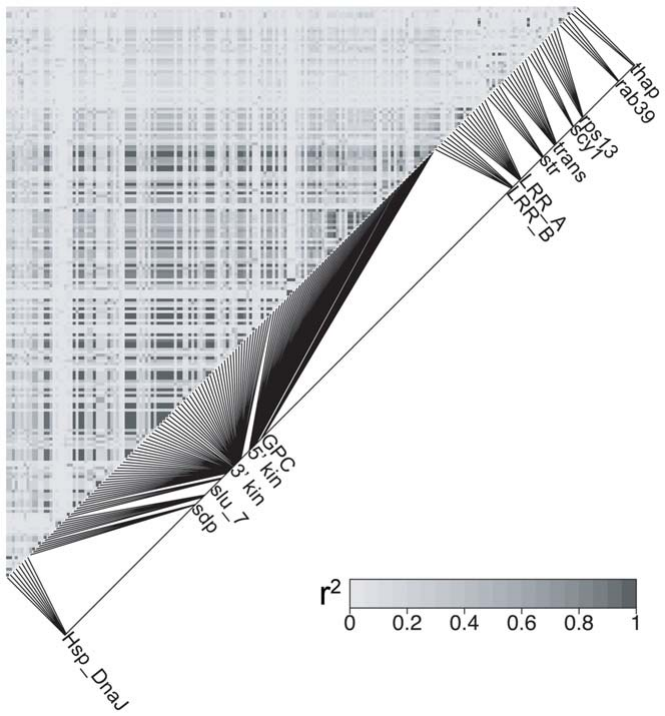
*HmB* linkage disequilibrium analysis. Pairwise estimates of linkage disequilibrium (r^2^) among 199 SNPs in the *HmB* locus (those with a rare allele frequency less than 10% were excluded) for combined *H. c. cordula* and *H. heurippa* population samples. Physical distance between sites is shown in the adjacent map.

The *HmB* locus was a significant outlier relative to the rest of the genome. We calculated a likelihood statistic that estimated the relationship of *H. heurippa* to the parental species, where positive values indicate sites linking *H. heurippa* with *H. m. melpomene* and negative values sites where *H. heurippa* is similar to *H. c. cordula*. The distribution of mean likelihood values across unlinked loci gives a distribution of expected values for the genome. Comparison of the 3′ *kinesin* region showed values that fell outside this distribution derived from unlinked markers (Figure 2; p<0.05), demonstrating that this genetic region has a significantly stronger association with *H. melpomene* than any other region of the *H. heurippa* genome. The *kin_2* region was also an outlier, but with a significantly stronger association with *H. cydno* (Figure 2; p<0.05), implying that the *kinesin* gene is in fact a chimera derived from two parental species. As *H. heurippa* is most closely allied to *H. c. cordula*, we analyzed linkage disequilibrium (LD) for these two species combined. Across the *HmB* region, the highest r^2^ values (p<0.001) were observed within the 3′ *kinesin* and nearby genes, with LD decaying in surrounding regions, suggesting a haplotype structure across the *kinesin* gene resulting from strong selection on wing pattern (Figure 3). Nonetheless, consistent with the wing patterning alleles being relatively ancient, there was no evidence for a reduction in diversity or deviation from neutrality that might indicate a recent selective sweep at 3′ *kinesin* (Table 1). Similarly, there was no evidence for adaptive amino acid substitution at this locus, with Ka/Ks ratios not significantly greater than 1 (p>0.05; Figure S1) and McDonald-Kreitman tests showing no deviation from neutrality (p>0.05). *H. heurippa* also had five private amino acid substitutions not observed in the other two species (Figure S2). Only one amino acid replacement was shared between the red-banded species, *H. heurippa* and *H. m. melpomene*, representing a putative causative site for this phenotype (Figure S2).

**Table 1:** Species nucleotide diversity for each locus.

	Gene	*H. cydno*			*H. melpomene*			*H. heurippa*		
		No of haplotypes	π/site	D_T_	No of haplotypes	π/site	D_T_	No of haplotypes	π/site	D_T_
	*Vldlr*	1	0,000001	n.a.	7	0,0020	0,6116	3	0,00062	0,2383
	*Sahase*	5	0,00160	0,5240	7	0,00183	−1,3627	3	0,00114	1,5955
	*Prs*	9	0,00294	0,6730	15	0,00496	0,0438	8	0,00215	−0,4204
	*tada 3*	5	0,00136	−1,2925	9	0,00317	0,1302	7	0,00832	2,4684*
	*Gapdh*	7	0,00383	1,1937	15	0,00736	1,4889	7	0,00318	0,4307
	*Atpsyn-β*	10	0,01283	1,1904	17	0,01440	0,2354	8	0,00835	−0,3142
	*ddx5*	4	0,00228	−0,1123	3	0,00087	0,2602	1	0,000001	n.a.
	*Trh*	14	0,00765	−0,1888	17	0,01263	−0,1233	6	0,00957	0,1551
	*chi-H*	14	0,00825	1,0933	9	0,00614	−0,3251	9	0,00526	−0,6954
	*hsp40*	7	0,00436	1,4149	4	0,00199	−0,0300	3	0,00102	−1,4354
	*glyRS*	7	0,00149	−1,5482	10	0,00342	0,8918	8	0,00290	−0,7074
	*ftsJ*	8	0,00199	−0,5603	9	0,00232	−1,5676	2	0,00054	1,0258
	*adipoR2*	11	0,00207	1,4880	8	0,00171	0,6996	4	0,00097	−0,3678
	*v-ATPase21*	4	0,00103	−0,9759	10	0,00635	0,9262	3	0,00151	−1,7341
	*hmg-coA*	20	0,01671	1,0175	17	0,00586	1,2900	12	0,01171	0,7124
	*srp68*	9	0,00370	0,2160	11	0,00470	0,5198	5	0,00137	0,2267
	*Psmd4*	5	0,00204	−1,7621	8	0,00296	0,2568	3	0,00031	−1,1407
	*Lm*	3	0,00141	0,2943	8	0,00343	0,6492	9	0,00422	0,1445
	*Aact*	12	0,00732	−0,5743	10	0,00629	0,6278	5	0,00465	1,5268
	*Hcl*	4	0,00086	0,4134	11	0,00371	2,2083*	4	0,00087	0,4572
	*bm18w*	12	0,00483	0,7025	18	0,00699	0,2591	9	0,00809	1,0862
	*Cmdh*	11	0,00870	−0,1991	12	0,00683	−0,0226	5	0,00815	−0,1837
	*Icdh*	11	0,01049	1,5849	14	0,00778	−1,1326	1	0,000001	n.a.
	*Can*	6	0,00163	1,6321	9	0,00288	1,3810	4	0,00226	2,3679*
	*Cad*	7	0,00368	−0,2354	9	0,01081	2,2204*	6	0,00321	−0,3692
	*Cat*	4	0,00193	1,6708	14	0,00182	0,0926	2	0,00169	2,0240*
	*hsp90*	11	0,00394	0,0157	12	0,01374	1,5819	8	0,01418	2,2808*
	*sae1*	8	0,00361	1,1135	15	0,01034	−0,6041	8	0,00583	1,1711
	*Acbp*	10	0,00775	0,7895	14	0,01131	−0,6584	5	0,00203	−1,0614
	*Hsp_DnaJ*	10	0,00480	1,7199	6	0,00165	0,5873	6	0,00209	1,4518
	*GPC_2*	8	0,00500	1,8017	13	0,00289	0,8262	2	0,00048	1,4302
	*Kin_1*	8	0,00306	−0,0132	7	0,00794	0,3965	1	0,000001	n.a.
	*Kin_2*	14	0,00830	1,4528	8	0,00221	0,3167	3	0,00081	1,1577
	*Kin_3*	5	0,00265	−0,3138	4	0,01146	1,0184	2	0,00070	−0,0861
	*Kin_4*	10	0,00390	0,3323	13	0,00698	−0,3579	6	0,00174	−0,3235
	*Kin 3′* ‡	20	0,00325	−0,1161	20	0,00153	−0,3144	15	0,00065	−0,6591
	*Slu7-1*	11	0,00323	−0,4943	14	0,00400	−0,3710	2	0,00023	−0,5915
	*Slu7-2*	8	0,00445	0,6944	7	0,00849	0,3442	5	0,00467	0,0289
	*Sdp*	8	0,00502	−0,6851	16	0,01171	−0,0100	9	0,00513	−1,8619*
	*THAP*	3	0,00183	0,5208	5	0,00275	0,2425	7	0,00247	0,6186
	*Rab39*	5	0,00131	−1,2597	5	0,00152	−1,2597	6	0,00208	0,5208
	*Rps13*	10	0,00386	0,4658	13	0,00547	0,4599	10	0,00418	1,4050
	*Scy1*	6	0,00174	0,2808	9	0,00278	−0,4204	4	0,00181	1,3853
	*Trans*	4	0,00101	1,1358	12	0,00413	−0,6951	17	0,00467	1,3088
	*Str*	4	0,00132	1,6831	14	0,00441	1,4754	12	0,00369	0,6887
	*LRR_B*	9	0,00187	−0,1818	11	0,00322	1,2727	12	0,00544	2,2229*
	*LRR_A*	11	0,00272	0,6116	12	0,00395	−0,5343	4	0,01068	0,3835

*: s indicate significance: * p<0.05; n.a.: not applicable due to lack of polymorphism.

**‡:** *Kin 3′* includes amplicons *Kin 8* to *Kin 14*. Haplotype numbers were determined over 20 sequences per species.

**Figure 4:**
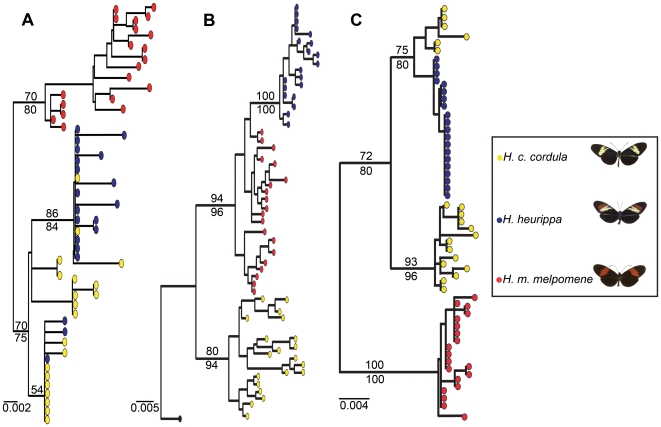
Gene genealogies for 3′ *kinesin* and its flanking regions. (A) sorting nexin (sdp); (B) 3′ *kinesin*; and (C) 5′ *kinesin* partial sequence (*kin*_2). Filled color circles represent alleles of each species. The 3′ *kinesin* tree is rooted with *H. numata* as outgroup (black). Numbers above and below the branches are bootstrap support values for likelihood and parsimony analyses respectively. (B) shows *H. heurippa* most closely related *to H. m. melpomene*, while (A,C), the genomic regions surrounding 3′ *kinesin*, show *H. heurippa* alleles more closely related to *H. c. cordula*. A similar tree topology was obtained from an amino acid alignment (data not shown).

An alternative hypothesis to adaptive introgression is that the *H. heurippa* pattern might be ancestral. However we could rule this out by reconstructing a rooted genealogy of the 3′ *kinesin* with either nucleotides or amino acid sequences (Figure 4B; data not shown for AA). Gene genealogies for *kin_2*, *sdp* and 3′ *kinesin* confirmed the results obtained with the SNP association tests (Figure 4). In the 3′ *kinesin*, *H. heurippa* was monophyletic and branched from within the *H. m. melpomene* clade, with *H. c. cordula* an outgroup to both species (Figure 4B, Table 2). In contrast, genomic regions surrounding 3′ *kinesin* showed *H. heurippa* alleles more closely related to *H. c. cordula* (Figure 4A and 4C; Table 2). Thus, the SNP analysis, LD patterns and gene genealogies revealed that a clearly delimited genomic portion of the *HmB* region closely relates *H. heurippa* with *H. m. melpomene*. This result is in contrast to the rest of the genome where no other gene showed a similar association.

**Table 2:** Net divergence among populations.

	Gene	Net divergence			Fixed differences			Shared polymorphisms		
		*h-m*	*h-c*	*c -m*	*h-m*	*h-c*	*c -m*	*h-m*	*h-c*	*c -m*
	*vldlr*	1.68	0.19	1.54	1	0	1	2	0	0
	*sahase*	0.91	1.01	0.25	0	0	0	0	0	1
	*prs*	1.68	4.80	2.83	0	0	0	6	1	5
	*tada 3*	2.79	2.91	0.33	0	0	0	2	2	4
	*gapdh*	2.46	0.43	2.23	0	0	0	5	4	3
	*atpsyn-β*	5.91	6.85	3.00	0	0	0	19	11	30
	*ddx*5	2.24	1.18	1.42	2	1	1	0	0	0
	*trh*	2.17	3.22	0.61	0	0	0	18	8	14
	*chi*-H	4.79	0.71	3.71	0	0	0	12	11	17
	*hsp40*	0.40	0.59	0.58	0	0	0	2	2	3
	*glyRS*	1.31	0.66	1.66	0	0	0	7	5	2
	*ftsJ*	5.03	3.31	3.34	3	2	1	0	0	1
	*adipoR2*	1.57	1.01	2.24	0	0	1	2	0	1
	*v-ATPase21*	2.95	0.58	2.73	0	0	0	6	0	0
	*hmg-coA*	2.13	7.83	11.19	0	0	0	4	30	11
	*srp68*	6.01	4.37	1.05	4	4	0	1	1	6
	*psmd4*	2.94	6.74	7.33	0	0	0	0	2	7
	*Lm*	0.87	2.06	1.22	0	0	0	4	1	0
	*aact*	8.99	9.52	13.70	4	0	2	0	7	11
	*hcl*	2.45	1.33	2.14	1	0	1	1	0	0
	*bm18w*	1.89	2.56	1.72	0	0	0	15	10	10
	*cmdh*	3.02	2.00	2.55	0	0	0	9	14	6
	*icdh*	12.57	14.69	3.42	9	9	0	0	0	15
	*cna*	1.75	0.27	1.02	0	0	0	1	3	2
	*cad*	5.68	0.51	6.67	0	0	0	4	8	3
	*cat*	6.64	1.85	7.11	3	0	4	0	0	0
	*hsp90*	12.80	10.95	8.33	1	0	1	11	7	4
	*sae1*	2.07	3.22	0.98	0	0	0	11	5	6
	*acbp*	4.45	9.02	7.21	0	0	0	6	6	21
	*Hsp_DnaJ*	0.81	1.11	0.63	0	0	0	1	1	2
	*GPC_2*	13.02	7.97	7.67	6	6	0	0	0	2
	*Kin_1*	4.86	3.26	1.86	4	2	1	0	0	1
	*Kin_2*	36.45	10.37	33.56	36	3	30	0	1	0
	*Kin_3*	3.39	2.56	2.18	2	0	1	0	0	2
	*Kin_4*	3.33	3.73	1.78	2	2	0	2	5	2
	*Kin 3′* ‡	17.07	34.78	22.02	13	28	14	4	6	6
	*Slu7-1*	1.51	1.42	0.89	1	1	0	0	0	1
	*Slu7-2*	9.90	10.33	6.05	0	0	8	9	5	1
	*Sdp*	4.05	1.22	2.78	0	0	0	3	3	2
	*THAP*	1.11	0.07	1.36	0	0	0	3	2	2
	*Rab39*	1.01	0.66	1.66	0	0	1	1	0	1
	*Rps13*	1.52	0.73	0.44	0	0	0	4	4	6
	*Scy1*	0.30	0.33	0.61	0	0	0	2	1	2
	*Str*	0.54	0.24	0.99	0	0	0	5	2	1
	*Trans*	1.71	0.97	4.82	0	0	1	7	1	0
	*LRR_B*	0.77	1.53	0.88	0	0	0	6	2	1
	*LRR_A*	0.88	1.57	0.73	0	0	0	4	2	2

**c:** *H. c cordula*; **m:** *H. m. melpomene*; **h:** *H. heurippa*.

**‡:** *Kin 3′* includes amplions *Kin 8* to *Kin 14*.

When the genealogy of 3′ *kinesin* was used to estimate divergence times, we found that *H. heurippa* alleles were derived from *H. m. melpomene* around 0.43 Mya (0.12–0.84) ago, subsequent to the splitting of the *H. c. cordula*/*H. m. melpomene* alleles at 2.82 Mya (1.03–5.22) ago. A coalescent expansion model [44], [45] (SSD>0.05 in all the cases) similarly suggested that *H. heurippa* haplotypes radiated more recently (∼0.385 Mya; 0.176–1.428) than either *H. m. melpomene* or *H. c. cordula* (∼2.055 Mya (1.175–4.219) and ∼2.745 Mya (1.584–4.489) respectively), giving a confirmation of the relative ages of the alleles found in each species, independent of tree topology. Thus, the *H. heurippa* 3′ *kinesin* alleles diverged subsequent to the split between *H. m. melpomene* and *H. c. cordula* at this locus.

**Figure 5:**
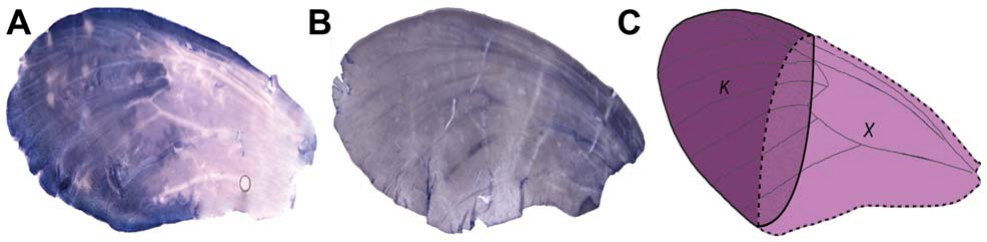
Expression pattern of *kinesin* in forewings. Of (A) *H. m. cythera* and (B) *H. cydno*. A similar expression pattern to that present in (A) is also observed in *H. m. rosina* forewings (Figure S3), consistent with the red band phenotype of these two races. The lack of any localized *kinesin* expression in *H. cydno* forewings is consistent with the absence of a red band in this species. (C) Model of how *kinesin* expression (K, solid line), might interact with an unknown gene (X, dotted line) to regulate forewing red band expression.

To examine the role of the putative *kinesin* gene in specification of wing pattern, we visualized spatial localization of *kinesin* transcripts in developing wings using *in situ* hybridization. In two red-banded forms *H. melpomene cythera* and *H. melpomene rosina*, a probe from exon 13 of the 3′ *kinesin* showed localization to the distal portion of the developing wing in early pupal stages (72–96 hrs after pupation; Figure 5A; Figure S3). No such spatial localization was seen either in individuals of *H. cydno* that do not express a red band phenotype (Figure 5B), nor in *H. melpomene* forewings treated with riboprobes for a different gene (Figure S3). This spatial localization suggests a model for pattern specification whereby the *kinesin* gene interacts with another as yet unidentified gene product to specify proximal and distal boundaries of the forewing band (Figure 5C), leading to upregulation of pigmentation genes such as *cinnabar* [46].

## Discussion

Homoploid hybrid speciation has been considered controversial in animals. We here provide the first molecular support for this hypothesis derived from sequence analysis of a gene region directly implicated in controlling a hybrid trait. *H. heurippa* was originally proposed as a hybrid species based on its unusual color pattern. The main evidence in support of this hypothesis are crossing experiments demonstrating experimental introgression of the *H. m. melpomene* red color forewing band into the *H. c. cordula* genomic background [35]. Such experiments demonstrate a plausible route for the origin of *H. heurippa*, and make a clear prediction: the region controlling the red forewing band should show a pattern of introgression from *H. melpomene* into *H. heurippa*. Here, we provide support for this hypothesis at a molecular level, by demonstrating a 6.5Kb region in the *HmB* locus that is introgressed from *H. melpomene* into *H. heurippa*.

The majority of SNPs (634) sampled in 29 coding genes, located on 17 of the 21 *H. melpomene* linkage groups, showed shared polymorphism among the three species (Figure 1A). *H. heurippa* and *H. c. cordula* shared fixed polymorphism relative to *H. m. melpomene* at only 8 SNPs, and there were no fixed SNPs in *H. heurippa* and *H. m. melpomene* relative to *H. c. cordula* (Figure 1A). This agrees with previous genetic data showing extensive allele sharing in the nuclear genome between the three species, but *H. heurippa* somewhat closer to *H. c. cordula* [40]. As we have argued previously [40], these data do not strongly support a hybrid speciation scenario, but are more consistent with either recent gene flow among the three species or shared ancestral polymorphism.

Here we have taken advantage of the recent cloning of *HmB*, the key locus underlying the speciation of *H. heurippa*. Our hypothesis derived from previous crossing experiments and sequence surveys is that the *H. heurippa* genome is most closely related to *H. cydno*, but with the introgression of the red forewing band, controlled by *HmB*, from *H. melpomene*. Here we directly test this hypothesis by sampling markers across the 721 Kb *HmB* locus [41]. From 13 genes evaluated in this region (comprising 24 molecular markers), we found a 6,493 bp region, corresponding to the 3′ end of the *kinesin* locus, where *H. heurippa* is strongly related to *H. melpomene* (Figure 1B). The likelihood values for species relationships at this locus differs significantly from that seen among unlinked genes, implying that this relationship cannot be explained by chance (Figure 2). The high long-range LD at *3*′ *kinesin* relative to the *H. c. cordula* genetic background is also expected under the introgression hypothesis (Figure 3). The pattern is comparable to that seen across the same region in a *Heliconius melpomene* hybrid zone, where long-range LD is observed between sites showing significant genotype-by-phenotype association [41]. A rather surprising observation is that across the *HmB* locus there is also shared variation between the three species, at sites interspersed between those generating a strong phylogenetic signal. These could be due to gene flow and recombination subsequent to speciation, recurrent mutations or alternatively a hybrid founding event for *H. heurippa* that transferred significant polymorphism from the parents to the hybrid species. In the data, there was no marked difference in the transition/transversion ratio among fixed and shared polymorphisms, which might be indicative of recurrent mutation (data not shown).

An alternative hypothesis to be considered is that *H. heurippa* pattern might be ancestral and have given rise to *H. melpomene* and *H. cydno* lineages that inherited different aspects of the ancestral wing pattern. However, this is not supported by the rooted gene genealogy for the 3′ *kinesin* that shows *H. heurippa* monophyletic, forming a well supported and derived clade within *H. melpomene* (Figure 4B). Furthermore, none of the dating approaches showed *H. heurippa* older than the other two species. Other genomic regions have shown a genealogical pattern whereby *H. heurippa* was similarly nested within an *H. cydno* clade, also arguing that *H. heurippa* is not an ancestral taxon [47].

In addition, *kinesin in situ* hybridizations on developing wing tissue (72–96h post-pupation) showed localized gene expression in the distal region of the wing, supporting a likely functional role in specification of the proximal boundary of the forewing band. In combination with previous analyses showing parallel differences in expression levels of the *kinesin* gene between color pattern races of both *H. melpomene* and *H. erato* [41], [48], these data strongly suggest that a regulatory change in the *kinesin* gene is functionally required for pattern determination. The *kinesin* gene appears to be chimeric, with the 3′ region derived from *H. m. melpomene* and the 5′ end more strongly related to *H. c. cordula*. Since crossing experiments suggest that introgression of the *HmB* allele from *H. melpomene* is sufficient to generate the *H. heurippa* pattern, the implication is that the functionally important sites are located at the 3′ end of the gene. We have identified one amino acid change and 11 synonymous changes shared between red-banded *H. melpomene* and *H. heurippa*, representing candidate functional sites for red band specification.

We also observed five amino acid differences between *H. melpomene* versus *H. heurippa* (Figure S2), perhaps reflecting adaptive change subsequent to formation of *H. heurippa*, although there was no significant evidence for selection on the locus (Figure S1). Perhaps more likely, these changes may represent fixation of nearly-neutral variation due to a population bottleneck during the origin of *H. heurippa*.

The implication of this gene in phenotypic control at *HmB* is also consistent with previous population genetic analysis of phenotypic races of *H. melpomene*, which showed a region of high genetic differentiation corresponding to a genomic region including the *kinesin* [41]. Members of the kinesin superfamily (KIFs) are key players in cellular functioning and morphology that interact with cargo molecules such as proteins, lipids or nucleic acids [49], [50]. In both vertebrates and invertebrates, *kinesin* molecules are implicated in pigment transport, however in *Heliconius melpomene* upregulation of pigment pathway genes occurs later in development relative to the localized *kinesin* expression observed here. This would suggest a likely upstream role in scale cell fate specification, rather than pigmentation *per se*.

Although adaptive introgression has recently been demonstrated at a molecular level, for example between species in the genus *Senecio* [19], *H. heurippa* is unusual in the fact that the hybrid trait contributes directly to reproductive isolation and hence speciation. We have also recently demonstrated that first generation backcross hybrids resembling *H. heurippa* also exhibit mate preferences very similar to that of wild *H. heurippa*. This implies that mate preferences could also have been produced by introgression in addition to color pattern [38]. A possible mechanism for this is suggested by the recent demonstration of a genetic association between the red band and male preference for red mates, in interspecific hybrids between *H. m. rosina* and *H. c. chioneus* (Merrill et al. pers. comm.). Thus, the derived color pattern and mate preferences of *H. heurippa* could potentially have arisen from introgression of the same gene or tightly linked genes.

Several cases of animal homoploid hybrid species have been recently proposed, such as *Rhagoletis* sp., *Lycaeides* sp., *Cottus gobio* group, cichlid fishes, *Xiphophorus clemensiae* and *Pogonomyrmex* sp. [17], [51] where ecological divergence, sexual selection or both promote reproductive isolation of a hybrid taxon. However, to our knowledge, this is the first time that molecular evidence for introgression has been established for an adaptive trait that also contributes directly to reproductive isolation and hence speciation. We feel that our results therefore represent the most convincing molecular evidence to date for homoploid hybrid speciation in animals. Similar molecular evidence also supports the hybrid origin of sunflower species in the genus *Helianthus*, although the pattern is very different in this case. Hybrid sunflower genomes are a mosaic of genomic blocks inherited from one or other parent [23], in contrast to *H. heurippa* which shares polymorphism with both parental species across most of the genome. Although mathematical simulation has suggested that the origin of *H. heurippa* probably involved an initial period of allopatry, during which the hybrid pattern became established [39], the contemporary genetic pattern supports our model of ‘hybrid trait speciation’ whereby localized introgression of key traits can promote the origin of hybrid species [21].

## Materials and Methods

### Specimen collection and DNA isolation

Butterflies were collected from Colombia and Venezuela: *H. m. melpomene* in Morcote (5°37′0.52″N; 72°18′0″W, Casanare-Colombia) and Chirajara (4°12′48″ N; 73°47′70″W, Cundinamarca-Colombia), *H. c. cordula* in San Cristobal (7°47′56″N; 72°11′56″W, Merida-Venezuela) and *H. heurippa* in Buenavista (4°10′30″N; 73°40′41″W, Meta –Colombia). Wings of 10 individuals of each species were removed and stored in glassine envelopes and are lodged in the Natural History Museum of the Universidad de los Andes. The bodies were preserved in 20% DMSO-0.25M EDTA salt saturated solution. DNA was isolated with DNeasy Blood & Tissue Kit (QIAGEN) following manufacturer's instructions. Quality of genomic DNA was confirmed by visualisation in a 0.8% agarose gel.

### Development, amplification, and sequencing of genetic markers

#### Single copy large exons

In order to develop new coding markers in *Heliconius*, we designed primers to amplify single copy large exon markers that were widely distributed across the *Heliconius* genome. We used the Basic Local Alignment Search Tool via nucleotide (BLASTN) to compare *Heliconius melpomene* genomic raw reads against unique genes (unigenes) of *Bombyx mori* [52]. Those unigenes showing homology with *H. melpomene* were subjected to BLASTN against *B. mori* whole genome shotgun (WGS) to reveal the location of introns [53]. Additionally, the program Spidey, freely available at http://www.ncbi.nlm.nih.gov/spidey/, was employed to confirm this information [43], [54]. With this knowledge in hand, introns and exons limits were determined in the *H. melpomene* contigs. To estimate the genomic location of selected markers we used the tool SilkMap [55], to locate genes on the silkworm chromosomes. Given highly conserved synteny between *B. mori* and *H. melpomene* [53], we could infer the putative chromosome location of markers in *Heliconius*. Identified *H. melpomene* contigs containing exons longer than 700 bp and located on different chromosomes were selected for PCR primer design using Primer 3 v.0.3.0 [56]. Two previously reported single copy large exons were also included [43]. We performed all PCR reactions in a 10 µL reaction volume containing 1× PCR buffer, 2.5 mM MgCl_2_, 200µM dNTPs, 1 µM each primer, 0.5 U Taq polymerase (QIAGEN) and 30–40 ng of DNA. The PCR cycling profile was 95°C for 5 min, 30 cycles of 95°C for 30 s, Tm for 45 s (see Table S2 for Tm values for each locus), 72°C for 1 min and final extension at 72°C during 15 min. Two microlitres of the PCR reaction were visualised in a 1% agarose gel to verify the success of PCR. The amplicons were cleaned up by ExoSAP-IT (USB Corp., Cleveland, OH). The BigDye Terminator Cycle Sequencing kit (PerkinElmer Applied Biosystems, USA) was used for direct sequencing using 24 cycles of denaturation at 96°C for 10 s, annealing at 50°C for 5 s, and extension at 60°C for 4 min. Sequencing reactions were cleaned up with Sephadex G50 (SIGMA) and analyzed with an ABI 3130*xl* DNA genetic analyzer (Applied Biosystems, USA).

#### Markers in the HmB locus region

A candidate region where the *HmB* locus is located in *H. m. melpomene* was previously described and annotated [41], [42]. It comprises seven BAC clones with a total length of around 721 kb [41], [42]. Here, we developed 24 PCR amplicons located in BAC clones AEHM-28L23, AEHM-21P16 and AEHM-27I5 (Table S1). PCR and clean up conditions were as described above. Direct sequencing was possible for 8 markers comprising only coding regions (Table S1). However, due to the presence of introns with considerable indel variation, the other 16 markers were cloned before sequencing (Table S1). PCR products were ligated into the pGEM-T easy vector (Promega). Five or more positive clones per individual were selected, re-amplified and again purified with ExoSAP-IT (USB Corp., Cleveland, OH) to identify distinct alleles. Direct sequencing of clones was performed as described above.

#### Sequence processing

Gene sequences were read, edited and aligned with Sequencher v4.6 (Gene Codes Corporation). For sequences resulting from direct sequencing, haplotypes reconstruction was conducted using DNAsp v4.90.1 [57] implementing the algorithm provided in PHASE [58]. Basically, PHASE assigns a probability of the correct inference of haplotype phase at every heterozygous position. PHASE simulations were repeated eight times for each locus, four without recombination and four with recombination. Each simulation was run with 5,000 iterations. In all cases, the most common output inferring haplotypes with >95% confidence was accepted. In the case of cloned products, aligned sequences of each individual were compared to discard PCR errors and false alleles caused by recombination in the cloning reaction. Purified alignments were translated to protein and checked for stop codons in MacClade v4.08 [59]. Sequences were deposited in GenBank under accession numbers GQ985506–GQ988326.

### Genetic analysis

#### SNP association statistic

Allele frequencies were calculated for each single nucleotide polymorphism (SNP) with rare allele frequency greater than 10% in the total sample of 60 haplotypes. With these numbers, a likelihood statistic was calculated to indicate the degree to which the *H. heurippa* SNP frequency was similar to *H. m. melpomene* relative to *H. c. cordula*.
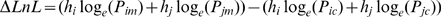
Where *h_i_* and *h_j_* are the number of *H. heurippa* individuals with *i* or *j* nucleotide respectively at a particular SNP position. The frequency of the nucleotide *i* or *j* in *H. m. melpomene* is represented by *p_im_* and *p_jm_*, respectively, and that in *H. c. cordula* by *p_ic_* and *p_jc_*. A positive value reflects *H. heurippa* being more similar to *H. m. melpomene*, while a negative value indicates association with *H. c. cordula*. Shared polymorphism at similar frequency in the three species, unique polymorphism in any one of the three species or a private allele in *H. heurippa* all give a value near to zero. For unlinked markers the average of these values was calculated to give a frequency distribution for the genomic background. Then, average values were similarly calculated in 1,000 bp windows across the *HmB* region and compared to the frequency distribution for unlinked markers. In order to determine if average likelihood values in the *HmB* region differ significantly from those in the genomic background, a confidence interval of 95% was established over the distribution of values for unlinked markers, by computing the 2.5 and 97.5 percentiles. Furthermore, using RSXL Excel [60], the statistical significance of outlier values was determined by resampling with replacement the likelihood averages for all 1,000 bp windows, with 40,000 repetitions.

#### Linkage disequilibrium analysis

When new polymorphisms are introduced by recent introgression at some time after species divergence, this should increase levels of linkage disequilibrium (LD) above the background within the region involved. We analyzed LD across the *HmB* locus for combined samples of *H. heurippa* and *H. c. cordula*. This calculation was made among 199 SNPs from the 24 genetic markers sampled in the *HmB* linked region. These SNPs were those where the minor allele frequency was greater than 10% (from 60 alleles) and thus, were considered as informative for the LD analysis. The LD computation was executed using the software MIDAS [61], which considers the distance between pairwise markers and does not assume that the genotypic phases are known. The resulting LD among the SNPs was visualized with the R package LDheatmap [62] plotting the r^2^ estimates versus physical distance.

#### Net divergence, nucleotide diversity, and protein evolution

For all the loci net divergence, fixed differences, shared polymorphisms and nucleotide diversity (π) were estimated with SITES [63]. Additionally, we calculated the number of substitutions per site for synonymous sites (Ks) and non-synonymous sites (Ka) in pairwise comparisons among the three species. The ratio Ka/Ks was determined in order to detect protein evolution in DNAsp v4.90.1 [57]. McDonald-Kreitman test [64] was also performed in DNAsp v4.90.1.

### Gene genealogies and time of introgression

In order to confirm the species relationships, genealogical topologies were reconstructed for three fragments within the *HmB* region, rooted for the 3′ *kinesin* (6,493 pb) using *H. numata* as outgroup and unrooted for 5′ *kinesin* partial sequence (*kin_2*; 1,100 pb) and sorting nexin (*sdp*; 402 pb) (Figure 4). Maximum Parsimony analysis was carried out in PAUP*v4.0b10 [65] using a heuristic search with TBR branch swapping; bootstrap values were calculated with 5,000 replicates using the same search conditions. Modeltest v3.7 [66] was used to determine the most appropriate model for nucleotide substitution based on corrected Akaike information criterion (AICc). For the 3′ *kinesin* data set Modeltest identified the HKY+I+G model, for 5′ *kinesin* the K81uf+I+G and for nexin the K80+I. Likelihood reconstructions were also made in PAUP*v4.0b10 [65] based on selected evolutionary models. Heuristic search and bootstrapping were carried out as for parsimony.

The 3′ *kinesin* genealogy was used to enforce a molecular clock hypothesis. When the likelihoods were compared, constant rate evolution was rejected (*x* ^2^ = 96.92, df = 48; P<0.001). Then a Bayesian framework, implemented in BEAST v1.4.8 [67], was employed to obtain an approximate time for the 3′ *kinesin* introgression. We applied the HKY+I+G model of evolution with four rate categories and assumed a relaxed lognormal clock. Based on the calibration proposed by Wahlberg et al. for Nymphalidae, with *Heliconius* and *Eueides* diverged from their common ancestor 18.4 Mya [68]. This date was used as a prior for a probabilistic calibration to determine the splitting time between *H. cydno* and *H. melpomene* alleles and between *H. heurippa* and *H. melpomene* alleles. The rest of the parameters were sampled keeping the default prior distributions. Two independent runs were implemented, with 50 million steps and burn-ins of 5,000,000. Tracer v1.4 was used to combine runs and observe parameter convergence [69]. Divergence time standard deviations were calculated from 95% confidence/credibility intervals using a normal approximation. We also computed the time of 3′ *kinesin* introgression under the assumption of species expansion. To perform this calculation, we first tested the fit of the observed mismatch distribution to the theoretical expectation as implemented in Arlequin v. 3.0 [70]. The calculations were made with a neutral mutation rate of ∼2.99×10^−10^ per base per generation for this region and 10 generations per year.

### Expression analysis

*kinesin* RNA *in situ* hybridizations were performed on *H. m. cythera*, *H. m. rosina* and *H. cydno* 72 to 96h pupal forewings. The specific races involved in the rest of the study were not available as live tissue for this analysis. A 303bp region of exon 13 in the *H. melpomene kinesin* gene was cloned into the vector pSPT19 (linearised with NheI). RNA probes were prepared with the DIG RNA labeling kit (SP6/T7) (Roche, Cat. 11 175 025 910) according to the manufacturer's instructions. Tissue fixation and in situ hybridization were carried out following a procedure modified from Ramos and Monteiro, 2007 [71].

## Supporting Information

Figure S1 Protein evolution analysis via Ka/Ks ratios. The distribution of ka/ks ratios for all genes for each species pair is shown. h: *H. heurippa*, m: *H. m. melpomene* and c: *H. c. cordula*. None of the comparisons had ka/ks >1, suggesting a lack of strong evidence for positive selection.
(0.54 MB PDF)

Figure S2 Protein sequence alignment of 3′ *kinesin*. The *kinesin* protein product from exon 9 to exon 14 is shown. Two representative sequences per species are shown. Residues with amino acid changes are highlighted. Polymorphic residues are indicated by a green asterisk, those residues where *H. heurippa* is different from *H. c. cordula* and *H. m. melpomene* are indicated by a blue asterisk and the red asterisk indicates one residue where *H. c. cordula* is different from *H. heurippa* and *H. m. melpomene*. We observed five amino acid differences between *H. melpomene* versus *H. heurippa*. This might reflect adaptive change subsequent to formation of *H. heurippa*, although there was no significant evidence for selection on the locus. Perhaps more likely however, these changes may represent fixation of nearly-neutral variation due to a population bottleneck during the origin of *H. heurippa*. Only one amino acid difference was found between *H. melpomene* and *H. cydno* in a residue also relating *H. melpomene* with *H. heurippa*. Although this amino acid replacement might be responsible for a structural protein change causing the red band, several intronic sites show a similar pattern and may have regulatory functions.
(0.31 MBPDF)

Figure S3 Controls for in situ hybridisations. (A) *Kinesin* expression in the forewing of the red-banded race *H. m. rosina* showing a distal expression of *kinesin* similar to that seen in *H. m. cythera*. The boundary of expression is more diffuse in this individual. The exact boundary position also varies between individuals (data not shown) most probably due to developmental stage. (B) Expression of gene *HMB000025* in the forewing of the red-banded race *H. m. cythera*. Unlike *kinesin*, *HMB000025* (a gene that is expressed in *H. melpomene* hingwings) does not show any localised expression pattern in the forewings. This indicates that the localization of expression for kinesin is probe-specific and not due to non-specific probe-trapping. (C) In situ control with no riboprobe.
(1.65 MB PDF)

Table S1 Genes and primer information for *HmB* linked markers.
(0.08 MB DOC)

Table S2 Genes and primer information for unlinked markers.
(0.09 MB DOC)
