# Supplementary material for: Genetic Evidence for Hybrid Trait Speciation in Heliconius Butterflies
Source: PLoS Genet. 2010 Apr 29;6(4):e1000930. doi: 10.1371/journal.pgen.1000930 (PMC2861694; doi:10.1371/journal.pgen.1000930)
Supplement: Figure S1 — Protein evolution analysis via Ka/Ks ratios. The distribution of ka/ks ratios for all genes for each species pair is shown. h: H. heurippa, m: H. m. melpomene and c: H. c. cordula. None of the comparisons had ka/ks >1, suggesting a lack of strong evidence for positive selection. (0.54 MB PDF) [file pgen.1000930.s001.pdf]

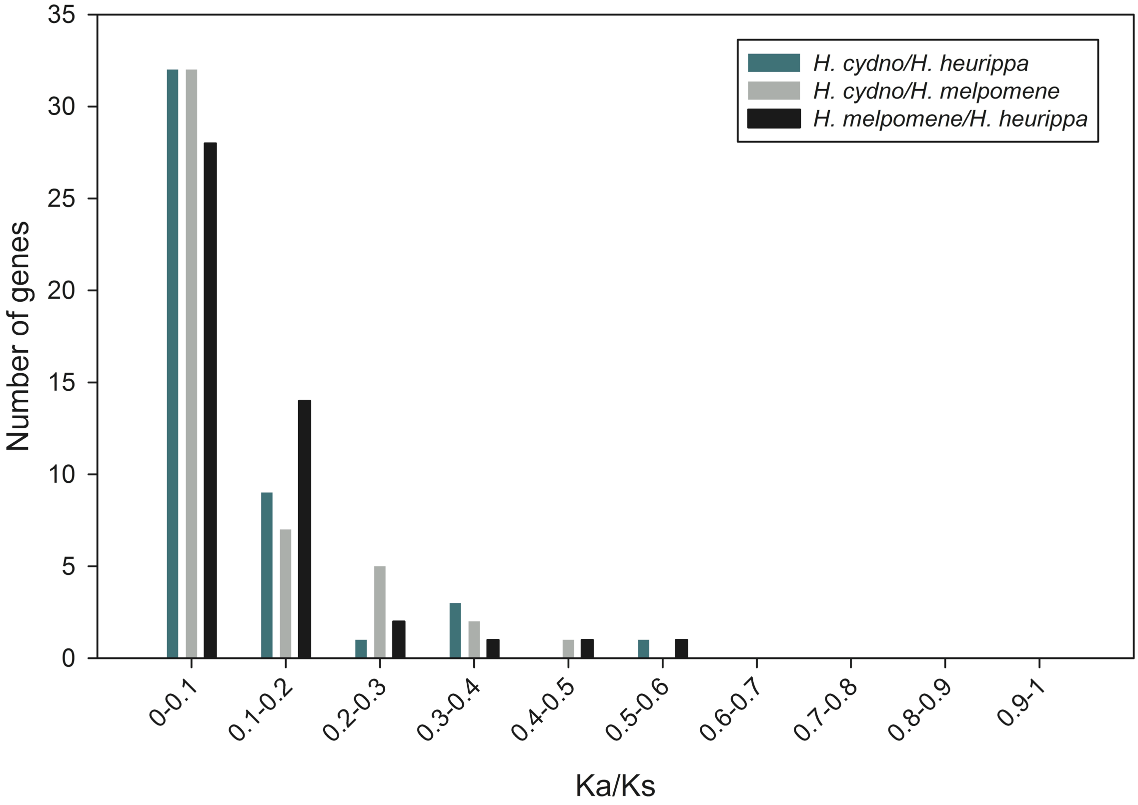

Figure S1. Protein evolution analysis via Ka/Ks ratios. The distribution of ka/ks ratios for all genes for each species pair is shown. h: *H. heurippa*, m: *H. m. melpomene* and c: *H. c. cordula*. None of the comparisons had ka/ks >1, suggesting a lack of strong evidence for positive selection.
