# Supplementary material for: Genetic Evidence for Hybrid Trait Speciation in Heliconius Butterflies
Source: PLoS Genet. 2010 Apr 29;6(4):e1000930. doi: 10.1371/journal.pgen.1000930 (PMC2861694; doi:10.1371/journal.pgen.1000930)
Supplement: Figure S2 — Protein sequence alignment of 3′ kinesin. The kinesin protein product from exon 9 to exon 14 is shown. Two representative sequences per species are shown. Residues with amino acid changes are highlighted. Polymorphic residues are indicated by a green asterisk, those residues where H. heurippa is different from H. c. cordula and H. m. melpomene are indicated by a blue asterisk and the red asterisk indicates one residue where H. c. cordula is different from H. heurippa and H. m. melpomene. We observed five amino acid differences between H. melpomene versus H. heurippa. This might reflect adaptive change subsequent to formation of H. heurippa, although there was no significant evidence for selection on the locus. Perhaps more likely however, these changes may represent fixation of nearly-neutral variation due to a population bottleneck during the origin of H. heurippa. Only one amino acid difference was found between H. melpomene and H. cydno in a residue also relating H. melpomene with H. heurippa. Although this amino acid replacement might be responsible for a structural protein change causing the red band, several intronic sites show a similar pattern and may have regulatory functions. (0.31 MBPDF) [file pgen.1000930.s002.pdf]

```
               1                 20                  40                  60                  80                 100
               •                  •                   •                   •                   •                   •
heurippa-1     AQSCIKELQSCMDERQATEADTMKELVEETKAITRDYYEIQIKILRDEMEQMEEEYESRLNKTVSASNGETPLKNMQLIKQLMTENAILEKYTAEKLARARA
heurippa-2     AQSCIKELQSCMDERQATEADTMEELVEETKAITRDYYEIQIKILRDEMEQMEEEYESRLNKTVSASNGETPLKNMQLIKQLMTENAILEKYTAEKLARARA
melpomene-1    AQSCIKELQSCMDERQATEADTMKELVEETKAITRDYYEIQIKILRDEMEQMEEEYESRLNKTVSASNGETPLKNMQLIKQLMTENAILEKYTAEKLARARA
melpomene-2    AQSCIKELQSCMDERQATEADTMKELVEETKAITRDYYEIQIKILRDEMEQMEEEYESRLNKTVSASNGETPLKNMQLIKQLMTENAILEKYTAEKLARARA
cydno-1        AQSCIKELQSCMDERQATEADTMKELVEETKAITRDYYEIQIKILRDEMEQMEEEYESRLNKTVSASNGETPLKNMQLIKQLMTENAILEKYTAEKLARARA
cydno-2        AQSCIKELQSCMDERQATEADTMKELVEETKAITRDYYEIQIKILRDEMEQMEEEYESCLNKTVSASNGETPLKNMQLIKQLMTENAILEKYTAEKLARARA
                                      *                                  *

                                120                 140                 160                 180                 200
                                  •                   •                   •                   •                   •
heurippa-1     EGEVQHLRACIDERDEKTDEDIPMKEDLVDLCDSEESDDDDSCNESLEPIFKREDMNRSGMIHQSAIIDENSFSSDHSDNSVLNKTYGTLKDENINNSNGND
heurippa-2     EGEVQHLRACIDERDEKTDEDIPMKEDLVDLCDSEESDDDDSCNESLEPIFKREDMNRSGMIHQSAIIDENSFSSDHSDNSVLNKTYGTLKDENINNSNGND
melpomene-1    EGEVQHLRACIDERDEKTDEDIPMKEDLVDLCDSEESDDDDSCNESLEPIFKREDMNRSRMIHQSAIIDENSFSSDHSDNSVLNKTYGTLKDENINNSNGND
melpomene-2    EGEVQHLRACIDERDEKTDEDIPMKEDLVDLCDSEESDDDDSCNESLEPIFKREDMNRSRMIHQSAIIDENSFSSDHSDNSVLNKTYGTLKDENINNSNGND
cydno-1        EGEVQHLRACIDERDEKTDEDIPMKEDLVDLCDSEESDDDDSCNESLEPIFKREDMNRSRMIHQSAIIDENSFSSDHSDNSVLNKTYGTLKDENINNSNGND
cydno-2        EGEVQHLRACIDERDEKTDEDIPMKEDLVDLCDSEESDDDDSCNESLEPIFKREDMNRSRMIHQSAIIDENSFSSDHSDNSVLNKTYGTLKDENINNSNGND
                                                                          *

                              220                 240                 260                 280                 300
                                •                   •                   •                   •                   •
heurippa-1     CTLETDNDVVAGEVVTTELNIDVHNNIEFKNHIKLSRETCLVENNDSFVNDKEKVLVSTLSRASNEDIVVDIDDKTKTNKNRNSFKDSSLETVDNNKPNTFV
heurippa-2     CTLETDNDVVAGEVVTTELNIDVHNNIEFKNHIKLSRETCLVENNDSFVNDKEKVLVSTLSRASNEDIVVDIDDKTKTNKNRNSFKDSSLETVDNNKPNTFV
melpomene-1    CTFETDNDVVAGEVVTTELNIDVHNNIEFKNHIKLSRETCLVENNDSFVNDKKKVLVSTLSRASNEDIVVDIDDKTKTNKNRNSFKDSSLETVDNNKPNTFV
melpomene-2    CTFETDNDVVAGEVVTTELNIDVHNNIEFKNHIKLSRETCLVENNDSFVNDKKKVLVSTLSRASNEDIVVDIDDKTKTNKNRNSFKDSSLETVDNNKPNTFV
cydno-1        CTFETDNDVVAGEVISTELNIDVHNNIEFKNHIKLSREACLVENNDSFVNDKKKVLVSTLSRASNEDIVVDIDDKTKTNKNRNSFKDSSLETVDNNKPNTFV
cydno-2        CTFETDNDVVAGEVVTTELNIDVHNNIEFKNHIKLSRETCLVENNDRFVNDKKKVLVSTLSRASNEDIVVDIDDKTKTNKNRNSFKDSSLETVDNNKPNTFV
                 *           **                      *       *     *

                            320                 340                 360                 380                 400
                              •                   •                   •                   •                   •
heurippa-1     NNIINSIKESNLSIQSNNSLASFEKLEEATKIDTGTNSNVTSFFNIKILKEKRNFFNYDDVLKSLEKPATVVKSKDKKIYFDNSNNNDTSNNDNEKIKIDDY
heurippa-2     NNIINSIKESNLSIQSNNSLASFEKLEEATKIDTGTNSNVTSFFNIKILKEKRNFFNYDDVLKSLEKPATVVKSKDKKIYFDNSNNNDTSNNDNEKIKIDDY
melpomene-1    NNIINSIKESNLSIQSNNSLASFEKLEEATKIDTGTNSNVTSFFNIKILKEKRNFFNYDDVLKSLEKPATVVKSKDKKIYFDNSNNNDTSNNDNEKIKIDDY
melpomene-2    NNIINSIKESNLSIQSNNSLASFEKLEEATKIDTGTNSNVTSFFNIKILKEKRNFFNYDDVLKSLEKPATVVKSKDKKIYFDNSNNNDTSNNDNEKIKIDDY
cydno-1        NNIINSIKESNLSIQSNNSLASFEKLEEATKIDTGTNSNVTSFFNIKILKEKRNFFNYDDVLKSLEKPATVVKSKDKKIYFDNSNNNDTSNNDNEKIKIDDY
cydno-2        NNIINSIKESNLSIQSNNSLASFEKLEEATKIDTGTNSNVTSFFNIKILKEKRNFFNYDDVLKSLEKPATVVKSKDKKIYFDNSNNNDSSNNDNEKIKIDDY
                                                                                                       *

                          420                 440                 460                 480                 500
                            •                   •                   •                   •                   •
heurippa-1     RSPSIIKEDVTNDYEPSMIKKLLGQSITNQNDYLNALQKIRTCDKKNDSIDLFEALDSPQACNKNKVNDDIENIRKSVQNVAIIQEDAIVCGNIEHNQEIVS
heurippa-2     RSPSIIKEDVTNDYEPSMIKKLLGQSITNQNDYLNALQKIRTCDKKNDSIDLFEALDSPQACNKNKVNDDIENIRKSVQNVAIIQEDAIVCGNIEHNQEIVS
melpomene-1    RSPSIVKEDVTNDYEPSMIKKLLGQSITNQNDNLNALQKIRTCDKKNDSIDLFEALDSPQACNKNKVNDDIENIRKSVQNVAIIQEDAIVCGNIEHNQEIVS
melpomene-2    RSPSIVKEDVTNHYEPSMIKKLLGQSITNQNDNLNALQKIRTCDKKNDSIDLFEALDSPQACNKNKVNDDIENIRKSVQNVAIIQEDAIVCGNIEHNQEIVS
cydno-1        RSPSIVKEDVTNDYEPSMIKKLLGQSITNQNDNLNALQKIRTCDKKNDSIDLFEALDSPQACNKNKVNDDIENIRKSVQNVAIIQEDAIVCGNIEHNQEIVS
cydno-2        RSPSIVKEDVTNDYEPSMIKKLLGQSITNQNDNLNALQKIRTCDKKNDSIDLFEALDSPQACNKNKVNDDIENIRKSVQNVAIIQEDAIVCGNIEHNQEIVS
                    *      *                   *
```

```
                        520                 540                 560                 580                 600
heurippa-1    NDELNNVEETNENKHVKEIVNDVDVESNKDNTIKKEEAIKISLEKSDDIEIVSNDETQLLINNTKSDNTSDEFENIYKDITTPRATEFDLLVSQEIKDTTID
heurippa-2    NDELNNVEETNENKHVKEIVNDVDVESNKDNTIKKEEAIKISLEKSDDIEIVSNDETQLLINNTKSDNTSDEFENIYKDITTPRATEFDLLVSQEIKDTTID
melpomene-1   NDELNNVEETNENKHVKEIVNDVDVESNNDNTIKKEEAIKISLEKSDDIEIVSNDETQLLINNTKSDNTSDEFENIYKDITTPRATEFDLLVSQEIKDTTID
melpomene-2   NDELNNVEETNENKHVKEIVNDVDVESNKDNTIKKEEAIKISLEKSDDIEIVSNDETQLLINNTKSDNTSDEFENIYKDITTPRATEFDLLVSQEIKDTTID
cydno-1       NDELNNVEETNENKHVKEIVNDVDVESNKDNTIKKEEAIKISLEKSDDIEIVSNDETQLLINNTKSDNTSDEFENIYKDITTPRATEFDLLVSQEIKDTTID
cydno-2       NDELNNVEETNENKHVKEIVNDVDVESNKDNTIKKEEAIKISLEKSDDIEIVSNDETQLLINNTKSDNTSDEFENIYKDITTPRATEFDLLVSQEIKDTTID
                                          *

                      620                 640                 660                 680                 700
heurippa-1    TTTNKETENEDLKYNLRQKSKMDKPKNETRRGKKVLESSPPKCEKSKTRKRNLRLRRQCGDNSEDGSKDIDEDTKKRDIEKAKLKDIINLQNEFSDVTLDKP
heurippa-2    TTTNNETENEDLKYNLRQKSKMDKPKNETRRGKKVLESSPPKCEKSKTRKRNLRLRRQCGDNSEDGSKDIDEDTKKRDIEKAKLKDIINLQNEFSDVTLDKP
melpomene-1   TTTNKETENEDLKYNLRQKSKMDKPKNETRRGKKVLESSPPKCEKSKTRKRNLRLRRQCGDNSEDGSKDIDEDTKKRDIEKAKLKDIINLQNEFSDVTLDKP
melpomene-2   TTTNNETENEDLKYNLRQKSKMDKPKNETRRGKKVLESSPPKCEKSKTRKRNLRLRRQCGDNSEDGSKDIDEDTKKRDIEKAKLKDIINLQNEFSDVTLDKP
cydno-1       TTTNNETENEDLKYNLRQKSKMDKPKNETRRGKKVLESSPPKCEKSKTRKRNLRLRRQCGDNSEDGSKDIDEYTKKRDIEKAKLKDIINLQNEFSDVTLDKP
cydno-2       TTTNKETENEDLKYNLRQKSKMNKPKNETRRGKKVLESSPPKCEKSKTRKRNLRLRRQCGDNSEDGSNDIDEDTKKRDIEKAKLKDIINLQNEFSDVTLDKP
                  *                *                                            *    *

                     720                 740                 760
heurippa-1    APDKEFKDIPSPEKNDNENLAPILGIQSCPAKTCITRTRRKLFTPRPEPLEESMEQST
heurippa-2    APDKEFKDIPSPEKNDNENLAPILGIQSCPAKTCITRTRRKLFTPRPEPLEESMEQST
melpomene-1   APDKEFKDIPSPEKNDNENLAPILGIQSCPAKTCITRTRRKLFTPRPEPLEESMEQST
melpomene-2   APDKEFKDIPSPEKNDNENLAPILGIQSCPAKTCITRTRRKLFTPRPEPLEESMEQST
cydno-1       APDKEFKDIPSPEKNDNENLAPILGIQSCPAKTCMTRTRRKLFTPRPEPLEESMEQST
cydno-2       APDKEFKDIPSPEKNDNENLAPILGIQSCPAKTCMTRTRRKLFTPRPEPLEESMEQST
                                                  *
```

**Figure S2. Protein sequence alignment of 3' *kinesin*.** The *kinesin* protein product from exon 9 to exon 14 is shown. Two representative sequences per species are shown. Residues with amino acid changes are highlighted. Polymorphic residues are indicated by a green asterisk (*), those residues where *H. heurippa* is different from *H. c. cordula* and *H. m. melpomene* are indicated by a blue asterisk (*) and the red asterisk (*) indicates one residue where *H. c. cordula* is different from *H. heurippa* and *H. m. melpomene.* We observed five amino acid differences between *H. melpomene* vs. *H. heurippa.* This might reflect adaptive change subsequent to formation of *H. heurippa*, although there was no significant evidence for selection on the locus. Perhaps more likely however, these changes may represent fixation of nearly-neutral variation due to a population bottleneck during the origin of *H. heurippa*. Only one amino acid difference was found between *H. melpomene* and *H. cydno* in a residue also relating *H. melpomene* with *H. heurippa*. Although this amino acid replacement might be responsible for a structural protein change causing the red band, several intronic sites show a similar pattern and may have regulatory functions.
