# Supplementary material for: Genetic Evidence for Hybrid Trait Speciation in Heliconius Butterflies
Source: PLoS Genet. 2010 Apr 29;6(4):e1000930. doi: 10.1371/journal.pgen.1000930 (PMC2861694; doi:10.1371/journal.pgen.1000930)
Supplement: Figure S3 — Controls for in situ hybridisations. (A) Kinesin expression in the forewing of the red-banded race H. m. rosina showing a distal expression of kinesin similar to that seen in H. m. cythera. The boundary of expression is more diffuse in this individual. The exact boundary position also varies between individuals (data not shown) most probably due to developmental stage. (B) Expression of gene HMB000025 in the forewing of the red-banded race H. m. cythera. Unlike kinesin, HMB000025 (a gene that is expressed in H. melpomene hingwings) does not show any localised expression pattern in the forewings. This indicates that the localization of expression for kinesin is probe-specific and not due to non-specific probe-trapping. (C) In situ control with no riboprobe. (1.65 MB PDF) [file pgen.1000930.s003.pdf]

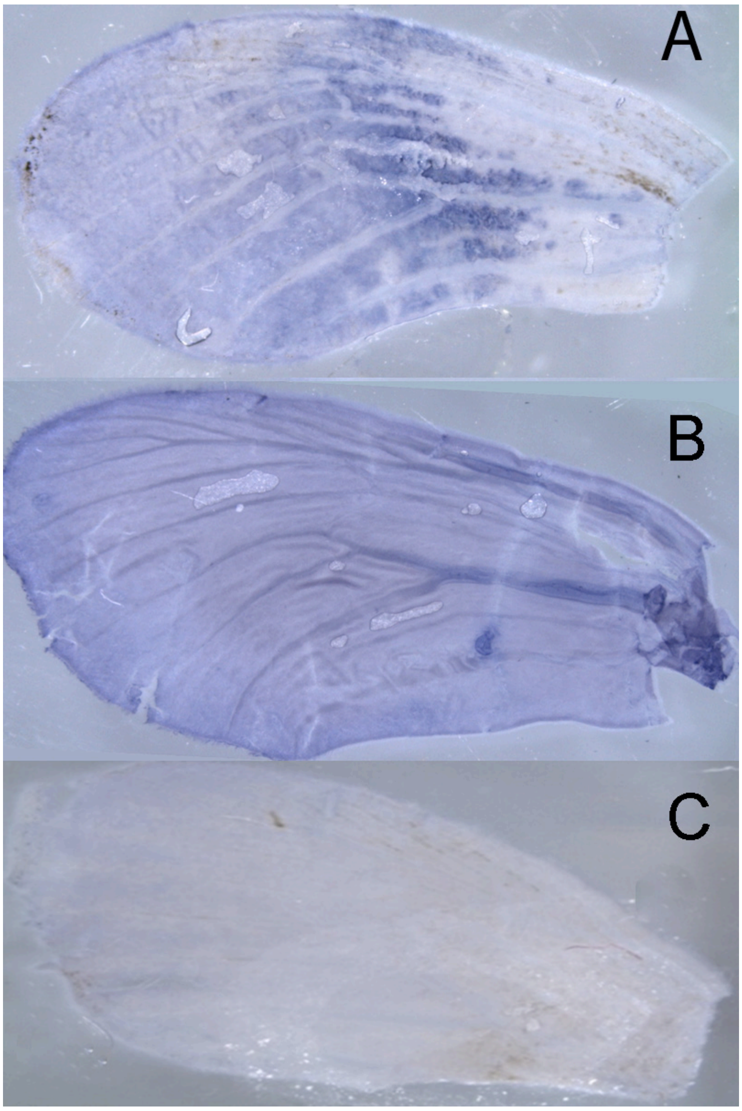

Figure S3. Controls for in situ hybridisations. A. *Kinesin* expression in the forewing of the red-banded race *H. m. rosina* showing a distal expression of *kinesin* similar to that seen in *H. m. cythera*. The boundary of expression is more diffuse in this individual. The exact boundary position also varies between individuals (data not shown) most probably due to developmental stage. **B.** Expression of gene *HMB000025* in the forewing of the red-banded race *H. m. cythera*. Unlike *kinesin, HMB000025* (a gene that is expressed in *H. melpomene* hingwings) does not show any localised expression pattern in the forewings. This indicates that the localization of expression for kinesin is probe-specific and not due to non-specific probe-trapping. **C.** In situ control with no riboprobe
