# Supplementary material for: Genetic Evidence for Hybrid Trait Speciation in Heliconius Butterflies
Source: PLoS Genet. 2010 Apr 29;6(4):e1000930. doi: 10.1371/journal.pgen.1000930 (PMC2861694; doi:10.1371/journal.pgen.1000930)
Supplement: Table S1 — Genes and primer information for HmB linked markers. (0.08 MB DOC) [file pgen.1000930.s004.doc]

**Table S1. Genes and primer information for *HmB* linked markers.**

| **Gene name** | **Abbreviation** | **Ps** | **Primer F (5' → 3')** | **Primer R (5' → 3')** | **Tm** | **Region amplified** |
| --- | --- | --- | --- | --- | --- | --- |
| Guanine nucleotide binding protein | *Hsp_DnaJ* | 614 | GTGCTCGGTGTCACAAGAGA | CTTCCAAAGCCATCTTGCAT | 52 | *** |
| G protein coupled receptor | *GPC_2* | 1100 | ACAGTTTTGTTAACCATGTT | TGATCTAAGTAGAAACCAAA | 48 | exon 6 intron 6 exon 7† |
| Kinesin | *Kin_1* | 549 | TTGAACCTAGGCCACCTGTAA | TCTGGACTTTGAGCAACATCA | 52 | exon 1 intron 1 exon 2† |
|  | *Kin_2* | 1100 | TTATGGGCACTGTCTCATCA | ATAGTGTCTTCTTCTATTAG | 48 | exon 2 intron 2 exon 3† |
|  | *Kin_3* | 443 | GCAAAGCAGGGAGGAGG | CCTTAACATATACATTGCCA | 50 | exon 3 intron3 exon4† |
|  | *Kin_4* | 1100 | TCTATAATGAAGGAATTTAT | CATAGTGATAGTGAATATAC | 48 | intron 4 exon 5† |
|  | *Kin_8* | 1404 | GATTGCACTTTGAATTGGTT | AAGACTGCAGTTCTTTTATA | 50 | exon 8 intron 8 exon 9† |
|  | *Kin_9* | 940 | TATAAAAGAACTGCAGT | AAGTTGCATATTCTTCA | 48 | exon 9 intron 9 exon 10† |
|  | *Kin_10* | 480 | TGGAGGAAGAATATGAAAGT | TGATCTATAAGTAAAATCAT | 48 | exon 10 intron 10 exon 11† |
|  | *kin_11* | 1400 | GCAACTGATGACTGAAAATGCT | TCTATACAAGCGCGCAAATG | 52 | exon11 intron 11 exon 12† |
|  | *Kin_12* | 1150 | ATAAATAAAATCTTCACTAC | TCGTTATTTTCTACAAGATA | 48 | intron11 exon 13† |
|  | *Kin_13* | 1267 | GTGCTATTATTGACGAAAAT | TAGCTCTCGGTGTTGTTATA | 50 | exon 13† |
|  | *Kin_14* | 1252 | TATAACAACACCGAGAGCTA | AGTTTTATGATACGAAGGTC | 52 | exon 13 intron 13 exon 14† |
| Step 2 splicing factor slu7 | *slu7-1* | 940 | GCGTGTGGCAGTATCTCAAA | CGTTTCTACAATCTTACAGT | 49 | exon 1 intron 1 exon2 intron 2 exon 3† |
|  | *slu7-2* | 1050 | GGCCGAACCGACTAAATTAC | ACAGCACTTGTATCCCCACT | 55 | exon 7 intron 7 exon 8 intron 8 exon 9† |
| Sorting nexin | *Sdp* | 402 | GAGGATACGACGGCAGAC | TCAGTCCTAAGCCATTCAAA | 55 | exon 1 intron 1 exon 2† |
| THAP protein domain | *THAP* | 551 | TACCAAAGCAATGATGAACG | AGCTCAACGACACTTTACAGGA | 56 | *** |
| RAB family member rab-39 | *Rab39* | 483 | TAATCGGTGACAGCACGGTA | TCTGCAAACATTCTAGCCTCTTC | 56 | *** |
| 40S ribosomal protein S13 | *Rps13* | 592 | AACCGCAGACGATGTTAAGG | AGAGGCGGTGCTAGACTCAT | 54 | *** |
| SCY1-like 2 protein | *Scy1* | 732 | ATGGTCGAAAACGGACAGAG | GGGTCAGGTCGTAATTCAGG | 56 | *** |
| Glycosyl transferase | *Trans* | 853 | TTGTGCTTTGACAGGACAGC | CCATAACATCGGCGGAAATA | 54 | *** |
| Strabismus | *Str* | 744 | ATGGCACCATTCCAAACAAG | TGACCTGCTTTCACCATCTG | 56 | *** |
| Leucine rich repeat A | *LRR-A* | 740 | TTGCGCTTAAACAAAAATGC | AAAACTGCCAATCCATGGTC | 53 | *** |
| Leucine rich repeat B | *LRR-B* | 973 | CGACGAAATGTTGTTTCACG | CCTCGATCCCTTCAACGATA | 56 | *** |

**Ps**: Product size in base pairs; **Tm:** Annealing temperature (°C); *******: See [39] for exon/intron information; †: Intron/exon

locations based on cDNA data.
