# Supplementary material for: Genetic Evidence for Hybrid Trait Speciation in Heliconius Butterflies
Source: PLoS Genet. 2010 Apr 29;6(4):e1000930. doi: 10.1371/journal.pgen.1000930 (PMC2861694; doi:10.1371/journal.pgen.1000930)
Supplement: Table S2 — Genes and primer information for unlinked markers. (0.09 MB DOC) [file pgen.1000930.s005.doc]

**Table S2.** Genes and primer information for unlinked markers.

| **Gene name** | **Abbreviation** | **LG** | **Ps** | **Primer F (5' → 3')** | **Primer R (5' → 3')** | **Tm** |
| --- | --- | --- | --- | --- | --- | --- |
| Very low density lipoprotein receptor | *vldlr* | 1 | 1008 | ATTGTTTCTGTTCCGCTGAT | TTTACATGTGTACAGTCTTT | 50 |
| S-adenosyl-L-homocysteine hydrolase | *sahase* | 1 | 835 | CATTAAACATGATATTAGAT | AATATCTGTAATGGTCAGGC | 49 |
| Phosphoribosyl-pyrophosphate synthetase | *prs* | 4 | 920 | TTGGAGAATAAGTATGAA | GAAATCAACTATAATTCT | 48 |
| Transcriptional adaptor 3 | *tada 3* | 5 | 740 | GGTGGAATGGGAGGAAGTGT | TTTGCGCGGGTAAATTTGTT | 54 |
| Glyceraldehyde-3-phosphate dehydrogenase* | *gapdh* | 5 | 702 | AARGCTGGRGCTGAATATGT | GWTTGAATGTACTTGATRAGRTC | 52 |
| H+ transporting ATP synthase beta subunit | *atpsyn-β* | 6 | 1057 | GCAGGAAAAGGCCAAGG | TCCATACCCAAGATGCAAT | 52 |
| Dead box popypeptide 5 | *ddx*5 | 7 | 716 | GAGATAACAGTTAGTGGCAAT | TCCTTCTCATGTTCTTCACA | 50 |
| Trachealess | *trh* | 9 | 735 | GATGCCACGTCCGTTAGAGA | CTATGAGCTTGTTGAATACC | 50 |
| Chitinase | *chi*-H | 9 | 1009 | TTTGGGATGGGGTGAGAGGA | CCAAGAGTGTCATTTG | 50 |
| Heat shock protein 40 Kda | *hsp40* | 10 | 560 | CACAATGGTCCAGGCGCT | TCACTGCCTTCTCTCTTGAA | 53 |
| Glycyl-tRNA synthetase | *glyRS* | 11 | 935 | AGACACGTTTTAACATTT | ATAGTGAATTCTCTAACTCT | 48 |
| Cell division protein | *ftsJ* | 12 | 822 | AACAAAATGGGAAAAACATC | TCCTTGTATGGGGGATTGAT | 50 |
| Adiponectin receptor 2 | *adipoR2* | 13 | 1011 | ACGTCGGCAAGGATGGG | CTAGAATGCAATGGAGGAG | 52 |
| ATP synthase 21 kDa proteolipid subunit | *v-ATPase21* | 13 | 724 | ATCACAAGACTTCTCCGTTT | TTTTCTTCTTAAGTTACTGG | 50 |
| 3-Hydroxy-3-Methyl-Glutaryl-CoA synthase | *hmg-coA* | 14 | 910 | CTTCTTTGGTCATCTATTTT | TATTGATACAACTAATG | 48 |
| Signal recognition particle 68 kDa protein | *srp68* | 15 | 891 | CGTCGTGTCACAGTGCGCCC | TACTTGTCACTCTTGCCCCA | 56 |
| Proteasome subunit 26S, non-ATPase 4, | *psmd4* | 15 | 922 | AAAATGGTATTGGAAAGTAC | GTAGACATAGCATTTCTGAT | 48 |
| Laminin | *lm* | 17 | 880 | TCCAGAATGCTATGCTTGTG | TTAATTCAGATGTAAGCTCT | 50 |
| Acetoacetyl-CoA thiolase | *aact* | 18 | 787 | TGATTTTAAGCTGCACAAGGA | ACTTACAATTTTTCAATCAT | 48 |
| Hairy cell leukemia protein | *hcl* | 18 | 778 | GCCGTAAAAGCAACCAC | AACATATAAATTACACCAAA | 48 |
| Bm18W | *bm18w* | 18 | 1247 | TAGGGTCGTAATTCTGGGTC | AAACAATATGACTGCCACCA | 52 |
| Cytosolic malate dehydrogenase | *cmdh* | 19 | 858 | CTTTTGTATCAAATTGCATCT | AATCTTTACAAACATCTAG | 48 |
| Isocitrate dehydrogenase | *icdh* | 20 | 984 | TATTTAGTCACTGGCCTAAG | ATCAAGGATAAATTAAT | 48 |
| Calcineurin A | *cna* | 20 | 1104 | GTGCCACCTTATTACGAT | TGGTGGCATCCTTTCATTT | 50 |
| CAD* | *cad* | 20 | 786 | GGN GTN ACN ACN GCN TGY TTY GAR CC | TTR TTN GGN ARY TGN CCN CCC AT | 57 |
| Catalase^‡^ | *cat* | 21 | 1131 | TCAAGACTGCGATTCAAACA | TGTCTTCAGTTTGTCCACT | 51 |
| Heat shock protein 90 Kda | *hsp90* | - | 1005 | AAATGCCAGAGGAAAAAATG | GATAAGGTCTTCACAGTTGT | 50 |
| SUMO-1 activating enzyme | *sae1* | - | 825 | CCAAATCCGCTTATGG | GAAGAAAAACATGTTATTAT | 48 |
| Acyl-CoA binding protein | *acbp* | - | 965 | TCCAGCATCTATCAAAAT | GTGCTCTTCTGGACCTGGAC | 50 |

* indicates those genetic markers obtained from [41] ; **LG:** Putative linkage group in *H. melpomene*; based on silkworm chromosomes synteny. **Ps:** Product size in base pairs; **Tm:** Annealing temperature (°C). ^‡^sex-linked marker.
